# Stem Cell Modeling of Core Binding Factor Acute Myeloid Leukemia

**DOI:** 10.1155/2016/7625827

**Published:** 2016-01-13

**Authors:** Federico Mosna, Michele Gottardi

**Affiliations:** Hematology, Department of Specialty Medicine, Ospedale Santa Maria di Ca' Foncello, Piazza Ospedale 1, 31100 Treviso, Italy

## Abstract

Even though clonally originated from a single cell, acute leukemia loses its homogeneity soon and presents at clinical diagnosis as a hierarchy of cells endowed with different functions, of which only a minority possesses the ability to recapitulate the disease. Due to their analogy to hematopoietic stem cells, these cells have been named “leukemia stem cells,” and are thought to be chiefly responsible for disease relapse and ultimate survival after chemotherapy. Core Binding Factor (CBF) Acute Myeloid Leukemia (AML) is cytogenetically characterized by either the t(8;21) or the inv(16)/t(16;16) chromosomal abnormalities, which, although being pathognomonic, are not sufficient *per se* to induce overt leukemia but rather determine a preclinical phase of disease when preleukemic subclones compete until the acquisition of clonal dominance by one of them. In this review we summarize the concepts regarding the application of the “leukemia stem cell” theory to the development of CBF AML; we will analyze the studies investigating the leukemogenetic role of t(8;21) and inv(16)/t(16;16), the proposed theories of its clonal evolution, and the role played by the hematopoietic niches in preserving the disease. Finally, we will discuss the clinical implications of stem cell modeling of CBF AML for the therapy of the disease.

## 1. Introduction: Leukemia-Initiating Cells in Core Binding Factor Acute Myeloid Leukemia

Core Binding Factor (CBF) Acute Myeloid Leukemia (AML) is cytogenetically defined by the presence of the t(8;21)(q22;q22) or the inv(16)(p13q22)/t(16;16)(p13;q22) [1], alterations that lead to the formation of fusion genes (*runt-related transcription factor 1 (RUNX)/RUNX1T1* and* CBFB/myosin heavy chain 11 (MYH11)*, resp.) that disrupt the signaling of the heterodimeric CBF complex with dominant prevalence. This ultimately results in impaired hematopoietic differentiation and clonal expansion [2]; as CBF translocations are not sufficient* per se* to determine overt leukemia, additional mutations act on these preleukemic clones, leading eventually to the emergence of the clinical disease.

**Figure 1:**
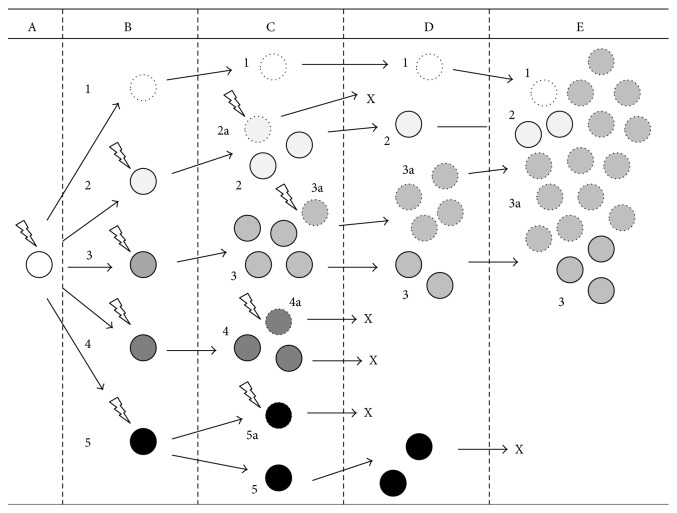
Simplified schematics of clonal evolution in leukemia. According to “clonal evolutionary theory,” soon after the initial leukemogenic event (here represented by a “lightning bolt”), that is, a first “class-2” mutation leading to expansion of a preleukemic clone (A), additional genetic mutations (again represented by “lightning bolts”) accumulate inside the cell population (B), creating a heterogeneous environment in which several distinct subclones compete for dominance (C). These subclones may later expand or spontaneously disappear (as here subclones 2a, 4, 4a, 5, and 5a do; clonal disappearance is marked by “X”) as a consequence of the selective pressure by intrinsic and extrinsic determinants (D). After the acquisition of “class-1” mutations, overt leukemia arises to clinical diagnosis (E) consisting of a dominant clone (3a in the Figure) and various subclones (1, 2, and 3).

In fact, even though originated by the evolution of a single genetically alterated cell, CBF AML, as most acute leukemias and cancers, loses its original homogeneity soon after its initial clonal expansion [3] (Figure 1). This is thought to be the result of the occurrence of random genetic mutations following DNA replication and alterations in the epigenetic control of the founding clone, as well as the consequence of impaired mechanisms of DNA repair [3]. At clinical diagnosis, the disease consists of heterogeneic clusters of cells that, besides sharing the defining chromosomal translocations and the common features of malignancy and loss of differentiation, widely differ from one another in terms of additional genetic lesions and function. Namely, only a strict minority of the leukemic population possesses the ability to propagate the disease [3, 4].

Based on their analogy to hematopoietic stem cells (HSC), that is, their role at the top of a hierarchical architecture, their immature characteristics, and their ability to recapitulate the disease with the morphological, immunophenotypic, and functional characteristics of the origin, these cells have been named “leukemia stem cells” (LSC) [3–5]. Despite these common features, however, crucial differences have soon been pointed out between HSC and LSC: among others, HSC are characterized by their vigilant control over proliferation and quiescence and the attentive preservation of their own genomic integrity, two features heavily damaged in LSC [4, 6]. Furthermore, the term LSC suggests their origin from HSC, which is probably not always the case in various types of leukemias. Therefore, we believe the term “leukemia-initiating cells” (L-IC) should be preferred to designate former LSC [4].

Given their definition based on a function, these cells are best identified, retrospectively, by means of a model of serial xenotransplantation of the human disease in immunodepressed mice. This model was first established in 1994 [5] by transplanting immunophenotypically sorted populations of AML blasts in NOD/SCID mice. Originally, L-IC have been identified as CD34+CD38− cells (i.e., by the combination of CD34 expression and the lack of CD38 and other markers of advanced commitment) in most samples of AML patients but with the notable exception of patients suffering from acute promyelocytic leukemia (APL) [5]. L-IC were coherently measured as a very strict minority of all leukemic blasts, accounting for 1 over 2.5 × 10^5^ cells. Later studies with more immunodepressed models, that is, NOD/SCID/IL2rg^−/−^ mice, have challenged these measures and proved that also CD34+CD38+ [7] and CD34−CD38− populations [8] hosted L-IC in approximately <50% of samples. Several reasons are thought to explain this discrepancy in L-IC measurements: (1) antibodies used during sorting (e.g., anti-CD38) may hamper the engraftment ability of CD38+ cells, thus abolishing the L-IC function of some CD34+CD38+ cells [7]; (2) the use of intravenous instead of the more sensitive intrafemoral injection to transfer the disease in mice may reduce the sensitivity of the test [9]; (3) residual immune rejection in NOD/SCID mice, granted by NK activity, may hamper the engraftment of human L-IC [7]; (4) interindividual variability, that is, disparity in L-IC numbers and immunophenotype among different patients, may alter measurements [10]; (5) L-IC engraftment may depend on species-specific interaction with the hematopoietic niches [9].

Research studies following Lapidot et al. [5], however, have not disproved either the original theory of AML as composed by a hierarchically organized population of blasts with different abilities or the presence of L-IC mainly in the CD34+CD38− subpopulation: in fact, L-IC could be identified in the CD34+CD38− group in samples of all AML patients (versus <50% of patients in the case of CD34+CD38+) [4], and as few as 1000 CD34+CD38− L-IC managed to serially recapitulate the disease upon xenotransplantation [11].

Subclonal genetic and functional heterogeneity is currently thought as a universal feature of cancer [12], even if this heterogeneity may vary significantly among cancer types [13] and during neoplastic progression [3]. In fact, L-IC might be seen as a dynamic functional entity [3], especially after chemotherapy and relapse, and a more ordered hierarchical system can evolve into a more homogeneous, stochastic model as cancer progresses [4]. Moreover, this implies that at a given time various genetically defined subclones may function as L-IC when tested in a xenotransplantation model; this has been demonstrated in ETV6-RUNX1 Acute Lymphoblastic Leukemia (ALL) [14]. L-IC property, as stemness at its core [6], may therefore be mostly a functional property of cells, thus suggesting a word of caution when addressing potential new therapies aimed at eradicating L-IC [3, 6].

## 2. Leukemogenesis as a Multistep Process Based on the Selective Evolution of Subclones

The recent advances in high-throughput sequencing methodologies have enabled scientists to fully map the genome of leukemic cells and to serially track the development of the subclones constituting the disease. This allowed confirming the “LSC theory” and demonstrating how L-IC share a common gene expression profile (GEP) with HSC [15]; the predominance of such a “stemness-related” GEP in AML strongly correlates with the adverse prognosis of patients [4, 15].

These data also established AML as characterized by an average of 13 different mutations* per* patient, with 5 of them in genes recurrently mutated in AML [16]. Computational analysis has been developed, allowing drawing the expansion of the competing subclones in leukemia as the cellular analogy of a species' evolutionary tree [3] (Figure 1). As many other cancer types, AML is therefore currently thought to evolve from a multistep process involving progressive genetic damage and increasing malignancy of the dominant clone [3, 9, 17, 18].

In fact, the existence of a preleukemic phase of AML was suggested by a series of historical works on asymmetric X chromosome inactivation, highlighting the existence of a stage of clonal hematopoiesis in most cases of AML [19]. This phase may present with the clinical features of a myelodysplastic syndrome or remain clinically silent until leukemia develops. In the case of CBF AML, the existence of a preleukemic phase was proved by a series of concomitant evidence: the pathognomonic CBF translocations could be found in normal HSC obtained from patients in remission [20–22]; a prolonged latency was consistently observed in experimental models between the occurrence of CBF translocations and the development of leukemia [23–25]; the finding of RUNX1-RUNX1T1/AML-ETO was constant at diagnosis and at relapse (similarly to CEBPA, DNMT3A, and IDH mutations, but differently from FLT3, N-RAS, and K-RAS mutations and WT1 overexpression, which are all thought to be later events in leukemogenesis) [10]; and finally, the long-term persistence of the molecular aberrant transcripts could be observed in some potentially cured patients [26, 27].

These observations are in line with the historical model of leukemogenesis proposed by Gilliland & Griffin as their “two-hit” theory [28]: the first step towards AML would consist in the acquisition of genetic alterations in “modulators of differentiation” (“class-2 mutations”) by the preleukemic clone, that is, CBF translocations in the case of CBF AML; the second step, then, would be the acquisition of mutations in “stimulators of proliferation,” disrupting cell-cycle controls (“class-1 mutations”), such as, in several cases of CBF AML, and activating mutations of KIT or RAS tyrosine kinases [24, 25, 29]. The occurrence of CBF translocation, originally, would hamper normal differentiation and facilitate the expansion of a clone primed for further genetic damage. In time, this would give rise to a variety of genetically defined subclones that compete for limited resources and with physiological hematopoiesis. Ultimately, the acquisition of clonal dominance, possibly by the acquisition of “class-1” mutations by one of these preleukemic subclones, ends the phase of clonal interference and gives rise to overt AML [3].

At the same time, the acquisition of L-IC function by the cells is not determined only by genetics, but it is rather the result of complex and still poorly clarified interactions between genetics, epigenetics, and interactions with the microenvironment. For instance, the fact that L-IC showed a CD34+/CD38− immature immunophenotype, similar to that of normal HSC, initially led to hypothesize that driver mutations leading to AML could only happen in HSC. Later evidence, though, showed how mixed lineage leukemia (MLL) fusion proteins, produced by translocations involving chromosome 11q23, are sufficient* per se* to confer self-renewal ability and L-IC properties to committed hematopoietic progenitors (HPP), as evidenced by the capacity of experimentally engineered MLL+ committed granulocyte/macrophage progenitors (GMP) to serially transfer leukemia to secondary recipient mice [30]. In fact, GEP of MLL-AF9-engineered GMP revealed the ectopic activation of stemness-related genes in addition to the characteristic genetic signature of these cells.

**Figure 2:**
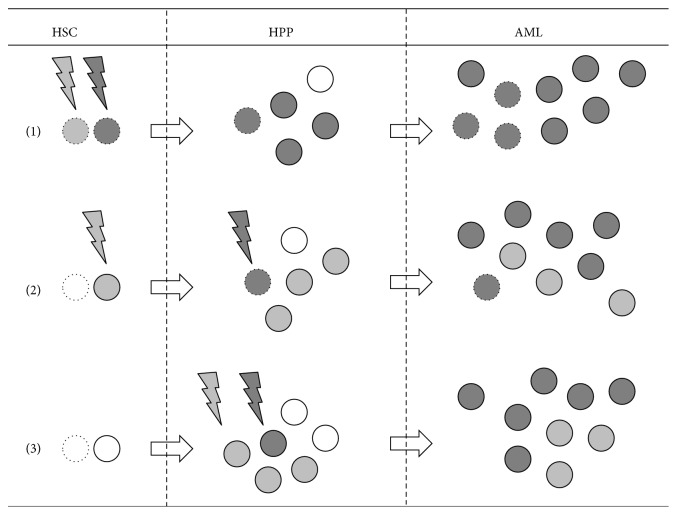
Proposed model for leukemogenesis. According to this model, AML arises following (at least) three different scenarios. In the first one (1) both “class-2” (represented as “light grey lightning bolts”) and “class-1” (“dark grey lightning bolts”) mutagenic events happen in the HSC (“white dotted circles”), thus creating a rapidly expanding clone endowed by some of the persisting physiological abilities of HSC, such as self-renewal ability (all cells with self-renewal abilities are represented as “dotted circles” in the figure). The resulting leukemia therefore contains more L-IC (represented by “grey dotted circles”), with consequences on the resistance to chemotherapy and the chance of relapse. In the second scenario (2) an initial “class-2” event gives rise to a preleukemic phase where different subclones (“light grey circles”) compete one another and with residual hematopoiesis (“white circles”) until the emergence of a dominant clone which benefits from self-renewal ability (“dark grey dotted circles”), conferred by an additional “class-1” mutation. This is the scenario thought to model leukemogenesis in the case of CBF AML. In the last scenario (3) leukemia arises from “class-2” and “class-1” events both happening in early committed HPP; leukemia therefore consists mainly of dysplastic HPP which are possibly more sensitive to chemotherapy and agents forcing differentiation.

Thus, at present at least three scenarios have been hypothesized to describe the early phases of leukemogenesis [10, 31] (Figure 2). In the first one, AML would evolve with “class-2” and “class-1” mutations both occurring in the most immature HSC compartment and as such involving cells already endowed with the fundamental features of stem cells (e.g., self-renewal ability, multipotency, and quiescence), which would be retained upon transformation. This scenario has been advocated as the case of DNMT3A-mutated NPM1-mutated AML [32] and is thought to model AML characterized by highly immature myeloperoxidase-negative nonlymphocytic blasts. In the second scenario, early “class-2” mutations occurring in the HSC compartment would be complemented by “class-1” mutations occurring at a later stage, in early committed, possibly CD33+ myeloid progenitors [10], and conferring long-term self-renewal ability. This would be the case of CBF AML [10] but perhaps also the case of several AML arising from a previous phase of myelodysplastic syndrome. Lastly, the third scenario would involve both types of driver mutations to happen in an already committed early myeloid progenitor. This would be the case of APL [10], where leukemic elements retain features of dysplastic promyelocytes.

This model of leukemogenesis hypothesizes possibly different effects by the same mutation when occurring in HSC or in early committed HPP. According to this model, quiescence and multipotency of HSC would endow their leukemic counterparts with an intrinsic better resistance to chemotherapy and allow the persistence of a larger number of L-IC after induction therapy. This could explain why a “stemness-related” GEP is prognostic for most cases of AML [15]. On the opposite, APL would be mostly devoid of L-IC [5] and would mostly benefit from differentiation-inducing agents, such as All-Trans Retinoic Acid or Arsenic Trioxide, which have generally failed against other types of AML. In the case of CBF AML, the peculiar chemosensitivity observed might also derive from the origin of their L-IC from early HPP primed by the presence of either RUNX1-RUNX1T1 or CBFB-MYH11.

## 3. Do RUNX1-RUNX1T1 and CBFB-MYH11 Fusion Genes Confer L-IC Function?

CBF as an heterodimer is essential for the development and homeostasis of definitive hematopoiesis [33, 34]; embryos homozygously lacking either the gene coding for its *α* subunit (called RUNX1 or, formerly, AML1) or *β* subunit (CBFB) die during embryogenesis without the development of HSC in the aorta-gonad-mesonephros (AGM) region [35, 36]. RUNX1 has a conserved role in hematopoiesis from* Nematodes* to humans [33, 37], while CBFB drives initial differentiation of hematopoietic precursors but shares part of RUNX1 roles in preserving the self-renewal ability of HSC [25]. In a recent zebrafish model RUNX1 was sufficient, in the absence of functional CBFB, for the emergence of HSC in the AGM region, but CBFB was required at a second step for the release of HSC from AGM into the circulation and thus for the formation of definitive hematopoiesis [38].

RUNX1-RUNX1T1 (formerly AML-ETO) lacks the RUNX1 transcription activation domain and as such acts as a dominant repressor for many RUNX1-responsive hematopoietic genes [33, 37]. CBFB-MYH11, on the other hand, shows a higher binding affinity than wild-type CBFB by acquiring a second RUNX-binding domain in MYH11 and dominantly inhibits regular CBF function by competing with the wild-type allele [33, 39]. In both cases, the occurrence of the translocations prevents hematopoietic differentiation of committed precursors [33] and determines the emergence of a preleukemic clone [23, 33, 37].

Recently, an experimental mouse model has been developed [40] in which the expression of the RUNX1-RUNX1T1 fusion gene was conditionally induced in hematopoietic cells, in order to resemble the progressive evolution and the mosaic expression pattern observed in human t(8;21) AML. This model was characterized by ineffective hematopoiesis coupled with the gradual expansion of committed GMP but not of long- and short-term repopulating HSC (LT- and ST-HSC) and common myeloid progenitors (CMP), ultimately leading to a syndrome mimicking human chronic myeloproliferative disorders [40]. Likewise, the expression of CBFB-MYH11 in hematopoietic cells results in leukemia only after a prolonged latency that can be shortened, in experimental models, by using mutagenesis strategies [41].

On the opposite, an alternatively spliced isoform of the RUNX1-RUNX1T1/AML1-ETO transcript, AML1-ETO9a, which includes an extra exon (9a) of the ETO gene, leads to the rapid development of acute leukemia in a mouse retroviral transduction-transplantation model [42]. Differently from their counterpart, AML1-ETO9a encodes a C-terminally truncated AML1-ETO protein of 575 amino acids, thus suggesting some kind of leukemia-inhibitory role for AML-ETO C-terminal portion.

In any case, the preleukemic clone originating from CBF translocations is primed for a second hit by the direct effects of both RUNX1-RUNX1T1 [43] and CBFB-MYH11 [44] on genes linked to DNA repair, cell-cycle, and self-renewal (among which those of the Notch pathway) [33, 43]. The “second hit” leading to overt AML may be represented by the acquisition of mutation in tyrosine kinases involved in cell cycle, such as KIT or RAS [24, 25, 29]. This is similar to what is observed in a pivotal AML model characterized by mutations in DNMT3A and NPM1 [32]: only DNMT3A mutations were present in sorted HSC; they often persisted in HPP after successful chemotherapy, and they preceded NPM1 mutations in the analysis of subclones. As such, DNMT3A was considered as one of the mutations determining expansion of the initial preleukemic clone, while the occurrence of mutation in NPM1 appeared as one of the later events determining clonal dominance and eventual development of AML.

## 4. Extrinsic Determinants of L-IC Function: The Role of the Hematopoietic Niches

Cancer is a complex microenvironment, in which intrinsic (i.e., genetics and epigenetics of the neoplastic cells) as well as extrinsic determinants (i.e., the interaction between neoplastic cells and their microenvironment) cooperate to determine overall clinical malignancy [4].

**Figure 3:**
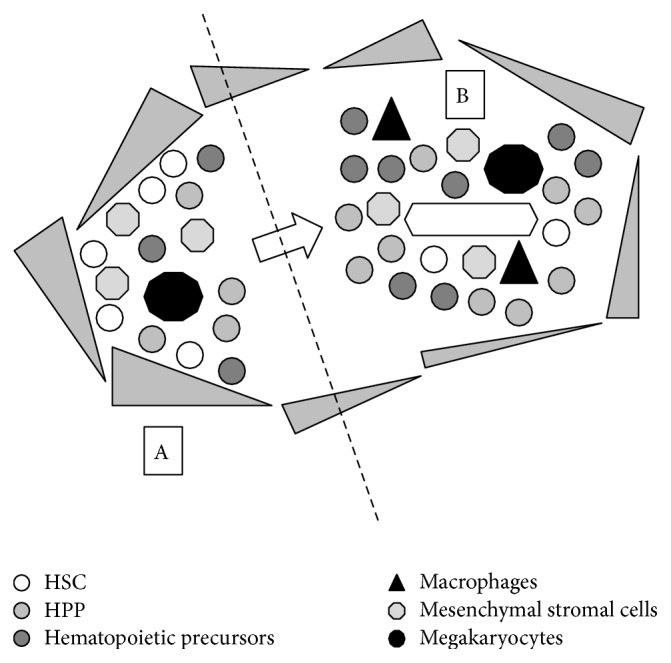
Simplified schematics of the endosteal and vascular hematopoietic niches. Two hematopoietic niches have been identified in the bone marrow, even though their nature as anatomical rather than functional entities is still a matter of debate. The first one, called the “endosteal niche” (A), is located near the endosteum and thought to harbour mainly quiescent HSC capable of extensive self-renewal. It is thought to be composed of osteoblasts (represented as a layer of “grey triangles” in the figure), mesenchymal stromal cells (“grey octagons”), megakaryocytes (“black decagons”), HSC (“white circles”), early committed HPP (“light grey circles”), and hematopoietic precursors at various degrees of commitment (“dark grey circles”). The second one, called the “vascular niche” (B), is located around the central vessel in the bone marrow and seems to harbour mainly rapidly proliferating HSC and HPP. It is composed by endothelial cells (boarding the central vessel), mesenchymal stromal cells (“grey octagons”), HSC (“white circles”), HPP (“light grey circles”), hematopoietic precursors at various degrees of commitment (“dark grey circles”), macrophages (“black triangles”), and megakaryocytes (“black decagons”). According to a theory, HSC would move from the endosteal to the vascular niche as they switch from a “resting” to a “proliferating” mode.

*In vivo*, the hematopoietic niches (i.e., the endosteal/osteoblastic and vascular niches) are involved in HSC physiology as primary extrinsic determinants (Figure 3). For instance, the expansion of osteoblasts in mice engineered with a osteoblast-specific, activated version of PTH/PTHrP receptors results in the expansion of its HSC reservoir via Jagged-1-Notch signaling, while their depletion determines the early exhaustion of HSC and the occurrence of aplastic anemia [45]. Similar effects are obtained if osteoblast numbers are increased through manipulation of the bone morphogenetic signaling pathway [46]. The interactions between HSC and other cells of the osteoblastic niche (osteoblasts [45, 46], mesenchymal stem cells [47, 48]), mediated by osteopontin [49, 50], c-kit [51, 52], the chemotactic SDF1-CXCR4 axis [48, 53], the Tie2-angiopoietin-1 tyrosine-kinase signaling [54], Notch [45, 55], and other molecules of cell adhesion, such as CD44 or CD123 [56–58], are all thought to be crucial in determining quiescence, and thus genomic preservation, of HSC [49, 50, 54]. In fact, HSC spontaneously differentiate when forced out of the niche by the digestion of cellular anchors [46], by the combination of Cyclophosphamide and Granulocyte Colony Stimulating Factor (G-CSF) [59], or by monoclonal antibodies [56, 58]. A model has been proposed by which early physiological differentiation of HSC corresponds to the movement from the endosteal niche (which is hypothesized to maintain stem cells in quiescence) to the vascular niche, where early expansion of HPP occurs [60] (Figure 3).

It is highly likely that these physiological properties of the hematopoietic niches are used by AML L-IC in their favour. In fact, AML L-IC, identified by their immunophenotype, have been maintained long-term* in vitro* by the coculture on a feeder layer of mesenchymal stromal cells, suggesting that the same may happen* in vivo* in HSC niches [61]. Moreover, in some animal models leukemia has been cured by inhibiting the interaction of blasts with the niche by the use of an anti-CD44 antibody [56, 57], which is currently being tested in experimental clinical trials. Recent studies have then described the ability of L-IC to invade physiological niches and, as previously hypothesized, to win the competition with HSC [62]. At the same time, others have suggested the capacity of L-IC to induce the formation of new niches and to colonize foreign organs [62]. This ability may differ among AML types: for instance, the expression of cell adhesion molecules and the incidence of extramedullary localization are maximal for acute monoblastic/monocytic leukemia [1]. At the same time, t(8;21) AML, but not inv(16) AML, characteristically presents with concomitant extramedullary granulocytic sarcoma in 8% of patients [1], thus implying some difference in its ability to engraft tissues originally devoid of hematopoietic niches. A possible explanation of this finding implies the Amyloid Precursor Protein (APP); the APP gene, in fact, was found upregulated in the case of complex karyotype AML and in those chromosomal rearrangements involving chromosome 21q21, where the gene is located [63]; a fraction of t(8;21) AML patients, then, has been linked to higher probability to develop granulocytic sarcoma and to lower long-term survival based on their level of APP expression [64]. APP is involved in cell adhesion and motility and increases* in vitro* matrix metalloproteinase-2 (MMP-2) expression by leukemic blasts [64]. We believe this may also enhance their extramedullary invasiveness* in vivo* and probably the chance to evolve as granulocytic sarcoma.

Besides their role in influencing stem cell properties, there are other ways by which hematopoietic niches might reduce chemosensitivity of leukemic blasts: for example, by inducing quiescence, thus preserving leukemic cells from cycle-dependent drugs [62]; by chemically altering the diffusion and effect of therapy by their mostly hypoxic conditions [65, 66]; by altering the conditions of the microenvironment, such as in preserving crucial amounts of Asparagine from the effect of L-ase treatment in ALL [67]. Overall, these studies all point out to the crucial role played by the microenvironment in influencing the development of leukemia.

## 5. The Clinical Viewpoint: Why Should Stem Cell Modeling of CBF AML Interest the Physician?

CBF AML patients have consistently showed enhanced survival after intensive chemotherapy, with long-term OS approaching 60% [68]. Even though these results have been linked to either peculiar sensitivity to high-dose Daunorubicin [69, 70] or Cytosine Arabinoside (HIDAC) [68, 71–81], no clear cutoff or dose increase has been identified to affect survival [79–81], and questions have arisen on whether the administration of repetitive consolidation cycles might be more important than the absolute dose intensity of HIDAC in determining survival [75, 76, 79–81].

As such, the biological basis explaining the intrinsic chemosensitivity of CBF AML, especially during consolidation therapy, remains still unclarified. As L-IC are thought to ultimately be responsible for leukemia relapse [4], it is possible that this should be attributable to the persistence of L-IC intrinsically more prone to chemotherapy because of their origin from an early committed HPP rather than HSC. The use of HiDAC in consolidation, furthermore, might be more apt to affect the protective abilities on L-IC of the hematopoietic niches. Finally, the persistence of preleukemic clones might explain why long-term disease control has been observed occasionally among patients with persisting molecular transcripts [20–22, 26, 27]; this theory is by no means negated by the possibility of successful antileukemia monitoring of CBF subclones by a restored immune system [26, 27].

Monoclonal antibodies are currently widely used in various fields of medicine to exert precise targeting of pivotal molecular pathways according to therapeutic needs. In the case of AML, CD33 has been chosen as a potential target based on its diffuse expression by myeloid progenitors and AML blasts but not by HSC. Gemtuzumab ozogamicin (GO), composed by a humanized monoclonal anti-CD33 antibody complexed with an antimitotic drug (Mitomycin), is the latest drug approved for clinical use and recently challenged in 3 wide European studies and one American trial [82]. Differently from the American trial, in all European studies the drug, used at dose ranging from 3 to 6 mg/m^2^ in both induction and consolidation therapy, has increased the OS chance in a randomized setting, without significantly increasing treatment mortality risk [83–85]; on the other hand, GO failed to increase results in patients affected by intermediate or high-risk AML [82–85]. Again, the efficacy of GO in CBF AML might have a biological explanation in differences of CBF AML L-IC as compared to other AML. In fact, if CBF AML L-IC indeed derive from the transformation of early committed HPP rather than HSC, they are likely to retain CD33 expression, thus becoming a target of GO therapy [10]. GO is currently withdrawn from the market after the disappointing results of the American trial [86]. As advocated by others [82, 87], we hope that this drug and its biological concept will be reconsidered for clinical use.

Autologous hematopoietic stem cell transplantation (ASCT) has been tested intensively in AML treatment as a tool to enhance consolidation therapy; in most cases, these attempts have proven unsuccessful with regard to long-term OS. A notable exception is CBF AML, where ASCT used after consolidation as part of first-line therapy improves DFS and OS in several studies [79, 88–90]; again, we believe the difference in L-IC biology might account for these results and the advantage observed after intensified first-line treatment [68, 72, 79, 80, 90, 91]. MRD monitoring currently provides a powerful tool to drive therapy intensification only in those high-risk patients actually needing it [79, 92, 93].

Once the bulk of disease is cleared by chemotherapy, a more specific treatment is needed to eradicate the disease by acting on residual L-IC surviving in protective hematopoietic niches. Monoclonal antibodies (mAb) may represent a potential approach to minimal residual disease eradication (reviewed in [58]). Among those targeting the interaction of L-IC and the hematopoietic niche, the anti-CD44 mAb H90 and the anti-CD123 mAb 7G3 hold the best promise in animal models, especially when used against low leukemic burden [56, 58]. Another approach might involve the targeting of molecular anchors. Plerixafor (AMD3100) is a small molecule targeting the fundamental SDF1-CXCR4 axis between HSC and cells of the hematopoietic niche [48, 94]. It might therefore mobilize AML L-IC from the niche, as observed after treatment in the case of CD34+ HSC, thus priming them to the effects of chemotherapy. The approach that failed in the past by using G-CSF combined with multiagent chemotherapy (e.g., FLAG, FLANG, FLAG-Ida, and GCLAC regimens) [94, 95] might find new* momentum* with Plerixafor.

## 6. Conclusions: From Stem Cell Modeling to Cure

Modern technologies are enabling at lower cost the complete genetic definition of AML patients. It is tantalizing to try and guess how this level of complexity will translate into the clinical practice: we can imagine a future where patients, following a debulking based on chemotherapy, will be treated individually on the basis of molecularly defined leukemia blueprints by a combination of molecular drugs. The results obtained by combining tyrosine-kinase inhibitors and chemotherapy in the treatment of Philadelphia-positive ALL provide an example of how the chance of cure can be ameliorated following a deeper molecular characterization of this disease [96]. In the case of KIT-mutated CBF AML, the use of Dasatinib in combination with intensive chemotherapy is currently tested by the National Cancer Institute (NCI) (ClinicalTrials.gov NCT01238211) and the German Acute Myeloid Leukemia Study Group (AMLSG) (ClinicalTrials.gov NCT00850382), while its use in the maintenance of patients achieving clinical remission with persistent molecular transcripts has recently failed to improve OS in another trial [97].

Often in science true progress comes from the contamination of ideas obtained from different fields of interest: the application of concepts derived from stem cell biology has already helped us to understand how leukemia originates and propagates; in the future, stem cell modeling of acute leukemia will lead us to a better way to treat the disease.

## References

[1] Arber D., Vardiman J., Brunning R., Swerlow S. H., Campo E., Harris N. L., etal (2008). Acute myeloid leukemia with recurrent genetic abnormalities. *WHO Classification of Tumours of Haematopoietic and Lymphoid Tissues*.

[2] Paschka P. (2008). Core binding factor acute myeloid leukemia. *Seminars in Oncology*.

[3] Greaves M., Maley C. C. (2012). Clonal evolution in cancer. *Nature*.

[4] Kreso A., Dick J. E. (2014). Evolution of the cancer stem cell model. *Cell Stem Cell*.

[5] Lapidot T., Sirard C., Vormoor J. (1994). A cell initiating human acute myeloid leukaemia after transplantation into SCID mice. *Nature*.

[6] Shackleton M. (2010). Normal stem cells and cancer stem cells: similar and different. *Seminars in Cancer Biology*.

[7] Taussig D. C., Miraki-Moud F., Anjos-Afonso F. (2008). Anti-CD38 antibody—mediated clearance of human repopulating cells masks the heterogeneity of leukemia-initiating cells. *Blood*.

[8] Taussig D. C., Vargaftig J., Miraki-Moud F. (2010). Leukemia-initiating cells from some acute myeloid leukemia patients with mutated nucleophosmin reside in the CD34^−^ fraction. *Blood*.

[9] Lane S. W., Gilliland D. G. (2010). Leukemia stem cells. *Seminars in Cancer Biology*.

[10] Walter R. B., Appelbaum F. R., Estey E. H., Bernstein I. D. (2012). Acute myeloid leukemia stem cells and CD33-targeted immunotherapy. *Blood*.

[11] Ishikawa F., Yoshida S., Saito Y. (2007). Chemotherapy-resistant human AML stem cells home to and engraft within the bone-marrow endosteal region. *Nature Biotechnology*.

[12] Marusyk A., Polyak K. (2010). Tumor heterogeneity: causes and consequences. *Biochimica et Biophysica Acta*.

[13] Quintana E., Shackleton M., Sabel M. S., Fullen D. R., Johnson T. M., Morrison S. J. (2008). Efficient tumour formation by single human melanoma cells. *Nature*.

[14] Greaves M. F., Wiemels J. (2003). Origins of chromosome translocations in childhood leukaemia. *Nature Reviews Cancer*.

[15] Gentles A. J., Plevritis S. K., Majeti R., Alizadeh A. A. (2010). Association of a leukemic stem cell gene expression signature with clinical outcomes in acute myeloid leukemia. *The Journal of the American Medical Association*.

[16] The Cancer Genome Atlas Research Network (2013). Genomic and epigenomic landscapes of adult de novo acute myeloid leukemia. *The New England Journal of Medicine*.

[17] Nowell P. C. (1976). The clonal evolution of tumor cell populations. *Science*.

[18] Merlo L. M. F., Pepper J. W., Reid B. J., Maley C. C. (2006). Cancer as an evolutionary and ecological process. *Nature Reviews Cancer*.

[19] Fialkow P. J., Singer J. W., Raskind W. H. (1987). Clonal development, stem-cell differentiation, and clinical remissions in acute nonlymphocytic leukemia. *The New England Journal of Medicine*.

[20] Nucifora G., Larson R. A., Rowley J. D. (1993). Persistence of the 8;21 translocation in patients with acute myeloid leukemia type M2 in long-term remission. *Blood*.

[21] Jurlander J., Caligiuri M. A., Ruutu T. (1996). Persistence of the AML1/ETO fusion transcript in patients treated with allogeneic bone marrow transplantation for t(8;21) leukemia. *Blood*.

[22] Miyamoto T., Nagafuji K., Akashi K. (1996). Persistence of multipotent progenitors expressing AML1/ETO transcripts in long-term remission patients with t(8;21) acute myelogenous leukemia. *Blood*.

[23] Corces-Zimmerman M. R., Hong W.-J., Weissman I. L., Medeiros B. C., Majeti R. (2014). Preleukemic mutations in human acute myeloid leukemia affect epigenetic regulators and persist in remission. *Proceedings of the National Academy of Sciences of the United States of America*.

[24] Shima T., Miyamoto T., Kikushige Y. (2014). The ordered acquisition of Class II and Class I mutations directs formation of human t(8;21) acute myelogenous leukemia stem cell. *Experimental Hematology*.

[25] Wang C. Q., Chin D. W. L., Chooi J. Y. (2015). Cbfb deficiency results in differentiation blocks and stem/progenitor cell expansion in hematopoiesis. *Leukemia*.

[26] Yin J. A. L., O'Brien M. A., Hills R. K., Daly S. B., Wheatley K., Burnett A. K. (2012). Minimal residual disease monitoring by quantitative RT-PCR in core binding factor AML allows risk stratification and predicts relapse: results of the United Kingdom MRC AML-15 trial. *Blood*.

[27] Perea G., Lasa A., Aventín A. (2006). Prognostic value of minimal residual disease (MRD) in acute myeloid leukemia (AML) with favorable cytogenetics [t(8;21) and inv(16)]. *Leukemia*.

[28] Gary Gilliland D., Griffin J. D. (2002). The roles of FLT3 in hematopoiesis and leukemia. *Blood*.

[29] Wichmann C., Quagliano-Lo Coco I., Yildiz Ö. (2015). Activating c-KIT mutations confer oncogenic cooperativity and rescue RUNX1/ETO-induced DNA damage and apoptosis in human primary CD34+ hematopoietic progenitors. *Leukemia*.

[30] Krivtsov A. V., Twomey D., Feng Z. (2006). Transformation from committed progenitor to leukaemia stem cell initiated by MLL-AF9. *Nature*.

[31] Jordan C. T., Guzman M. L. (2004). Mechanisms controlling pathogenesis and survival of leukemic stem cells. *Oncogene*.

[32] Shlush L. I., Zandi S., Mitchell A. (2014). Identification of pre-leukaemic haematopoietic stem cells in acute leukaemia. *Nature*.

[33] Goyama S., Mulloy J. C. (2011). Molecular pathogenesis of core binding factor leukemia: current knowledge and future prospects. *International Journal of Hematology*.

[34] Speck N. A., Gilliland D. G. (2002). Core-binding factors in haematopoiesis and leukaemia. *Nature Reviews Cancer*.

[35] Okuda T., van Deursen J., Hiebert S. W., Grosveld G., Downing J. R. (1996). AML1, the target of multiple chromosomal translocations in human leukemia, is essential for normal fetal liver hematopoiesis. *Cell*.

[36] Wang Q., Stacy T., Miller J. D. (1996). The CBFbeta subunit is essential for CBFalpha2 (AML1) function in vivo. *Cell*.

[37] Lam K., Zhang D.-E. (2012). RUNX1 and RUNX1-ETO: roles in hematopoiesis and leukemogenesis. *Frontiers in Bioscience*.

[38] Bresciani E., Carrington B., Wincovitch S. (2014). CBF*β* and RUNX1 are required at 2 different steps during the development of hematopoietic stem cells in zebrafish. *Blood*.

[39] Kelly L. M., Gilliland D. G. (2002). Genetics of myeloid leukemias. *Annual Review of Genomics and Human Genetics*.

[40] Cabezas-Wallscheid N., Eichwald V., de Graaf J. (2013). Instruction of haematopoietic lineage choices, evolution of transcriptional landscapes and cancer stem cell hierarchies derived from an AML1-ETO mouse model. *EMBO Molecular Medicine*.

[41] Castilla L. H., Garrett L., Adya N. (1999). The fusion gene Cbfb-MYH11 blocks myeloid differentiation and predisposes mice to acute myelomonocytic leukaemia. *Nature Genetics*.

[42] Yan M., Kanbe E., Peterson L. F. (2006). A previously unidentified alternatively spliced isoform of t(8;21) transcript promotes leukemogenesis. *Nature Medicine*.

[43] Alcalay M., Meani N., Gelmetti V. (2003). Acute myeloid leukemia fusion proteins deregulate genes involved in stem cell maintenance and DNA repair. *Journal of Clinical Investigation*.

[44] Mandoli A., Singh A. A., Jansen P. W. T. C. (2014). CBFB-MYH11/RUNX1 together with a compendium of hematopoietic regulators, chromatin modifiers and basal transcription factors occupies self-renewal genes in inv(16) acute myeloid leukemia. *Leukemia*.

[45] Calvi L. M., Adams G. B., Weibrecht K. W. (2003). Osteoblastic cells regulate the haematopoietic stem cell niche. *Nature*.

[46] Zhang J., Niu C., Ye L. (2003). Identification of the haematopoietic stem cell niche and control of the niche size. *Nature*.

[47] Méndez-Ferrer S., Michurina T. V., Ferraro F. (2010). Mesenchymal and haematopoietic stem cells form a unique bone marrow niche. *Nature*.

[48] Van Overstraeten-Schlögel N., Beguin Y., Gothot A. (2006). Role of stromal-derived factor-1 in the hematopoietic-supporting activity of human mesenchymal stem cells. *European Journal of Haematology*.

[49] Nilsson S. K., Johnston H. M., Whitty G. A. (2005). Osteopontin, a key component of the hematopoietic stem cell niche and regulator of primitive hematopoietic progenitor cells. *Blood*.

[50] Stier S., Ko Y., Forkert R. (2005). Osteopontin is a hematopoietic stem cell niche component that negatively regulates stem cell pool size. *Journal of Experimental Medicine*.

[51] Driessen R. L., Johnston H. M., Nilsson S. K. (2003). Membrane-bound stem cell factor is a key regulator in the initial lodgment of stem cells within the endosteal marrow region. *Experimental Hematology*.

[52] Heissig B., Hattori K., Dias S. (2002). Recruitment of stem and progenitor cells from the bone marrow niche requires MMP-9 mediated release of Kit-ligand. *Cell*.

[53] Dar A., Kollet O., Lapidot T. (2006). Mutual, reciprocal SDF-1/CXCR4 interactions between hematopoietic and bone marrow stromal cells regulate human stem cell migration and development in NOD/SCID chimeric mice. *Experimental Hematology*.

[54] Arai F., Hirao A., Ohmura M. (2004). Tie2/angiopoietin-1 signaling regulates hematopoietic stem cell quiescence in the bone marrow niche. *Cell*.

[55] Duncan A. W., Rattis F. M., DiMascio L. N. (2005). Integration of Notch and Wnt signaling in hematopoietic stem cell maintenance. *Nature Immunology*.

[56] Jin L., Hope K. J., Zhai Q., Smadja-Joffe F., Dick J. E. (2006). Targeting of CD44 eradicates human acute myeloid leukemic stem cells. *Nature Medicine*.

[57] Krause D. S., Lazarides K., von Andrian U. H., van Etten R. A. (2006). Requirement for CD44 in homing and engraftment of BCR-ABL-expressing leukemic stem cells. *Nature Medicine*.

[58] Majeti R. (2011). Monoclonal antibody therapy directed against human acute myeloid leukemia stem cells. *Oncogene*.

[59] Passegué E., Wagers A. J., Giuriato S., Anderson W. C., Weissman I. L. (2005). Global analysis of proliferation and cell cycle gene expression in the regulation of hematopoietic stem and progenitor cell fates. *The Journal of Experimental Medicine*.

[60] Yin T., Li L. (2006). The stem cell niche in bone. *Journal of Clinical Investigation*.

[61] Ito S., Barrett A. J., Dutra A. (2015). Long term maintenance of myeloid leukemic stem cells cultured with unrelated human mesenchymal stromal cells. *Stem Cell Research*.

[62] Lane S. W., Scadden D. T., Gilliland D. G. (2009). The leukemic stem cell niche—current concepts and therapeutic opportunities. *Blood*.

[63] Baldus C. D., Liyanarachchi S., Mrózek K. (2004). Acute myeloid leukemia with complex karyotypes and abnormal chromosome 21: amplification discloses overexpression of APP, ETS2, and ERG genes. *Proceedings of the National Academy of Sciences of the United States of America*.

[64] Jiang L., Yu G., Meng W., Wang Z., Meng F., Ma W. (2013). Overexpression of amyloid precursor protein in acute myeloid leukemia enhances extramedullary infiltration by MMP-2. *Tumor Biology*.

[65] Parmar K., Mauch P., Vergilio J.-A., Sackstein R., Down J. D. (2007). Distribution of hematopoietic stem cells in the bone marrow according to regional hypoxia. *Proceedings of the National Academy of Sciences of the United States of America*.

[66] Gilbert L. A., Hemann M. T. (2010). DNA damage-mediated induction of a chemoresistant niche. *Cell*.

[67] Iwamoto S., Mihara K., Downing J. R., Pui C.-H., Campana D. (2007). Mesenchymal cells regulate the response of acute lymphoblastic leukemia cells to asparaginase. *The Journal of Clinical Investigation*.

[68] Grimwade D., Hills R. K. (2009). Independent prognostic factors for AML outcome. *Hematology/the Education Program of the American Society of Hematology*.

[69] Fernandez H. F., Sun Z., Yao X. (2009). Anthracycline dose intensification in acute myeloid leukemia. *The New England Journal of Medicine*.

[70] Löwenberg B., Ossenkoppele G. J., van Putten W. (2009). High-dose daunorubicin in older patients with acute myeloid leukemia. *The New England Journal of Medicine*.

[71] Grimwade D., Walker H., Oliver F. (1998). The importance of diagnostic cytogenetics on outcome in AML: analysis of 1,612 patients entered into the MRC AML 10 trial. The Medical Research Council Adult and Children's Leukaemia Working Parties. *Blood*.

[72] Grimwade D., Hills R. K., Moorman A. V. (2010). Refinement of cytogenetic classification in acute myeloid leukemia: determination of prognostic significance of rare recurring chromosomal abnormalities among 5876 younger adult patients treated in the United Kingdom Medical Research Council trials. *Blood*.

[73] Slovak M. L., Kopecky K. J., Cassileth P. A. (2000). Karyotypic analysis predicts outcome of preremission and postremission therapy in adult acute myeloid leukemia: a Southwest oncology group/Eastern cooperative oncology group study. *Blood*.

[74] Mrózek K., Prior T. W., Edwards C. (2001). Comparison of cytogenetic and molecular genetic detection of t(8;21) and inv(16) in a prospective series of adults with de novo acute myeloid leukemia: a Cancer and Leukemia Group B study. *Journal of Clinical Oncology*.

[75] Byrd J. C., Mrózek K., Dodge R. K. (2002). Pretreatment cytogenetic abnormalities are predictive of induction success, cumulative incidence of relapse, and overall survival in adult patients with de novo acute myeloid leukemia: results from cancer and leukemia group B (CALGB 8461). *Blood*.

[76] Marcucci G., Mrózek K., Ruppert A. S. (2005). Prognostic factors and outcome of core binding factor acute myeloid leukemia patients with t(8;21) differ from those of patients with inv(16): a Cancer and Leukemia Group B study. *Journal of Clinical Oncology*.

[77] Appelbaum F. R., Kopecky K. J., Tallman M. S. (2006). The clinical spectrum of adult acute myeloid leukaemia associated with core binding factor translocations. *British Journal of Haematology*.

[78] Cairoli R., Beghini A., Grillo G. (2006). Prognostic impact of *c-KIT* mutations in core binding factor leukemias: an Italian retrospective study. *Blood*.

[79] Mosna F., Papayannidis C., Martinelli G. (2015). Complex karyotype, older age, and reduced first-line dose intensity determine poor survival in core binding factor acute myeloid leukemia patients with long-term follow-up. *American Journal of Hematology*.

[80] Löwenberg B. (2013). Sense and nonsense of high-dose cytarabine for acute myeloid leukemia. *Blood*.

[81] Paschka P., Döhner K. (2013). Core-binding factor acute myeloid leukemia: can we improve on HiDAC consolidation?. *American Society of Hematology. Education Program*.

[82] Rowe J. M., Löwenberg B. (2013). Gemtuzumab ozogamicin in acute myeloid leukemia: a remarkable saga about an active drug. *Blood*.

[83] Burnett A. K., Hills R. K., Milligan D. (2011). Identification of patients with acute myeloblastic leukemia who benefit from the addition of gemtuzumab ozogamicin: results of the MRC AML15 trial. *Journal of Clinical Oncology*.

[84] Castaigne S., Pautas C., Terré C. (2012). Effect of gemtuzumab ozogamicin on survival of adult patients with de-novo acute myeloid leukaemia (ALFA-0701): a randomised, open-label, phase 3 study. *The Lancet*.

[85] Burnett A. K., Russell N. H., Hills R. K. (2012). Addition of gemtuzumab ozogamicin to induction chemotherapy improves survival in older patients with acute myeloid leukemia. *Journal of Clinical Oncology*.

[86] Petersdorf S. H., Kopecky K. J., Slovak M. (2013). A phase III study of gemtuzumab ozogamicin during induction and post-consolidation therapy in younger patients with acute myeloid leukemia. *Blood*.

[87] Castaigne S. (2013). Why is it so difficult to use gemtuzumab ozogamicin?. *Blood*.

[88] Fernandez H. F., Sun Z., Litzow M. R. (2011). Autologous transplantation gives encouraging results for young adults with favorable-risk acute myeloid leukemia, but is not improved with gemtuzumab ozogamicin. *Blood*.

[89] Nakasone H., Izutsu K., Wakita S., Yamaguchi H., Muramatsu-Kida M., Usuki K. (2008). Autologous stem cell transplantation with PCR-negative graft would be associated with a favourable outcome in core-binding factor acute myeloid leukemia. *Biology of Blood and Marrow Transplantation*.

[90] Döhner H., Estey E. H., Amadori S. (2010). Diagnosis and management of acute myeloid leukemia in adults: recommendations from an international expert panel, on behalf of the European LeukemiaNet. *Blood*.

[91] Byrd J. C., Ruppert A. S., Mrózek K. (2004). Repetitive cycles of high-dose cytarabine benefit patients with acute myeloid leukemia and inv(16)(p13q22) or t(16;16)(p13;q22): results from CALGB 8461. *Journal of Clinical Oncology*.

[92] Paietta E. (2012). Minimal residual disease in acute myeloid leukemia: coming of age. *Hematology/American Society of Hematology. Education Program*.

[93] Grimwade D., Freeman S. D. (2014). Defining minimal residual disease in acute myeloid leukemia: which platforms are ready for ‘prime time’?. *Blood*.

[94] Mohty M., Ho A. D. (2011). In and out of the niche: perspectives in mobilization of hematopoietic stem cells. *Experimental Hematology*.

[95] Becker P. S. (2004). Growth factor priming in therapy of acute myelogenous leukemia. *Current Hematology Reports*.

[96] Fielding A. K. (2015). Treatment of Philadelphia chromosome-positive acute lymphoblastic leukemia in adults: a broader range of options, improved outcomes, and more therapeutic dilemmas. *Hematology*.

[97] Boissel N., Renneville A., Leguay T. (2015). Dasatinib in high-risk core binding factor acute myeloid leukemia in first complete remission: a French Acute Myeloid Leukemia Intergroup trial. *Haematologica*.

